# The Recovery of Damaged Pine Forests in an Area Formerly Polluted by Nitrogen

**DOI:** 10.1100/tsw.2001.451

**Published:** 2001-12-08

**Authors:** Kestutis Armolaitis, Vidas Stakenas

**Affiliations:** Lithuanian Forest Research Institute, Girionys, Lithuania

**Keywords:** reduced nitrogen pollution, Scots pine stands, refoliation, ground vegetation, Luvisols, Arenosols, acidity, nitrate, chemical composition

## Abstract

An area in Lithuania containing coniferous stands of Scots pine and Norway spruce that were dead or damaged due to nitrogen pollution by a nitrogen fertilizer plant (JV Achema) was found to have expanded between 1974 and 1989 to a distance of 20 to 25 km northeast of the plant in the direction of prevailing winds. Over the last 10 years, when nitrogen pollution by the plant had decreased, a clear process of recovery of the damaged ecosystems could be observed. The following features of this process as it occurred in damaged Scots pine stands are discussed: (1) refoliation (or decreased defoliation) of damaged trees, where a clear positive trend could be observed; (2) changes in the species composition and in the covering by ground vegetation, where small changes and indication of less-nitrophilous species coverage could be detected; and (3) chemical and acidity changes in Luvisols and Arenosols, where a significant decrease could be seen especially concerning nitrate concentrations.

## INTRODUCTION

At the beginning of the 1970s, large plants for producing nitrogen fertilizers for agricultural development were built in the European part of the former Soviet Union, specifically in Russia (Novgorod and Dzerzhinsk), the Ukraine (Rovno and Severodonetsk), Lithuania (Jonava), and Belorussia (Grodno). The impact that pollutants from these nitrogen fertilizer plants had on forests of highly developed West European countries such as Sweden has been rather insignificant (partly due to its being episodic)[1]. In the vicinity of the huge nitrogen fertilizer plants in the former Soviet Union, however, the decline or mortality of forests at distances of up to 20 to 25 km in the direction of prevailing winds could be observed[2]. At lesser distances, changes in soil chemical properties and subsequent soil acidification were ascertained[2,3].

The present article summarizes the condition of forest ecosystems in the vicinity of a nitrogen fertilizer plant (JV Achema) in Lithuania that was put into operation in 1965. The plant was surrounded by forests, and society viewed their resulting decline in quite negative terms. This led to a variety of studies on the degradation of the forest ecosystem[2,3,4,5,6,7,8]. An obvious recovery of the damaged forest ecosystems has been observed during the reduction in air pollution that has occurred during the past 10 years. The following features of this process in the Scots pine stands will be discussed: (1) recovery of vitality by the trees; (2) changes in plant species composition and in ground vegetation; and (3) changes in forest litter and in soil-chemical parameters.

## EXPERIMENTAL METHODS AND PROCEDURES

Investigations were conducted at different distances of up to 22 km to the northeast of the JV Achema plant, in the direction of prevailing winds. This plant is situated in central Lithuania (55°05' north, 24°20' east). In the forests surrounding it there is a prevalence of Scots pine (*Pinus sylvestris* L.), 39%; birch (*Betula pendula* Roth.), 22%; and Norway spruce (*Picea abies* [L.] Karst.), 20%. These stands have an average age of 50 years[9].

The forest coverage is 39%, and the sandy forest soils are classified mainly as Arenosols or Luvisols.

During the period from 1974 to 1989, when forests (mostly Scots pine and Norway spruce stands) were declining[10] in an area ranging from 20 to 25 km in the direction of prevailing winds from the JV Achema plant, annual emissions from the plant amounted to some 15,000 to 40,000 tonnes (Fig. 1). The greatest part of these pollutants consisted of dust from mineral fertilizers (38%) and of CO (27%), as well as $SO_2$ (13%), $NO_x$ (10%), and $NH_3$ (10%) gases[11]. From 1990 to 1992, the process of forest decline halted when the annual emission amounts decreased to a level of 11,000 to 12,000 tonnes. Later, in the period from 1993 to 2000, the amount of pollutants decreased still further to only about 5,000 to 7,000 tonnes annually. Closing of the most environmentally polluting shops and the use of natural gas instead of oil has led to a changed composition of pollutants such that CO (67%), $NO_x$ (9 to11%), and $NH_3$ (7 to 11%) gases prevail.

From 1997 to 2000, air pollution by $NO_2$ and $SO_2$ gases was monitored by use of passive diffusion samplers[12]. These were placed in 10 to 15 study locations 2 m above the ground two to three times during the vegetation period, for periods of 30 to 40 days each. The diffusion samplers were prepared by and analysed at the Institute of Physics in Vilnius.

The condition of the 50- to 100-year-old Scots pine stands was studied from 1995 to 2000 at seven permanent observation plots (POPs) established at distances of 10 to 22 km from the plant. In each POP assessments were made of the defoliation and discoloration of 170 to 280 examples of predominant, dominant, and codominant trees (rated according to the Kraft system)[13].

Ground-vegetation studies of 60- to 80-year-old *Vaccinio-myrtillosum* Scots pine stands in 16 POPs at distances of 10 to 12 km and 20 to 22 km from the plant were carried out during June of 1988 and 1999. In each POP, the floristic composition and the covering of individual plant species in eight vegetation-sample squares was assessed.

Assessment of phytocenotic changes was based on the mean weighted value of a corresponding ecological factor ($E_L$ = light, $E_N$ = amount of nitrogen in the soil, and $E_R$ = soil acidity):

$$E_f = \sum \frac{N_i \cdot p_i}{P},$$

where $E_f$ is the ecological factor, $N_i$ the indicator value of the ecological factor for *i*th species[14], $p_i$ is covering by the *i*th species, and *P* the overall covering by all the layer species. The data obtained were compared with ground vegetation data for Scots pine stands of corresponding age in nonindustrial areas of Lithuania[15].

In 1979, forest soils were studied in the cutting area of dead Scots pine stands (Luvisols, 0.2 km from JV Achema) and in stands of pine that survived (Arenosols, located at distances of 2 and 22 km from the plant). According to data from earlier years, chemical parameters of the Luvisols 0.2 km from the plant and of Arenosols 2 km from the plant were subjected to changes due to air pollution, whereas Arenosols that were located 22 km from the plant could be regarded as relatively unpolluted[2]. The samples of forest litter and of mineral horizons (from layers 0–5, 5–10, 10–20, 25–35, and 45–55 cm deep) were collected in September in either five or ten replicates. The forest litter mass was determined in the field. The following measures for the forest soil samples were analysed: pH in a 1 M KCl suspension, $NO_3$-N by ionometer; $NH_4$-N (in a 1% $K_2SO_4$ suspension), mobile phosphorus ($P_2O_5$) (in a 0.2 M HCl suspension), and mobile S (in a 1 M KCl suspension). Except pH all were analysed by colorimetric methods. $Al^{3+}$ was analysed in a 1 M KCl suspension via the titration method[16].

The statistical significance of the differences obtained was assessed by use of Student's *t*-test.

## RESULTS AND DISCUSSION

### Reduction of Air Pollution

During the vegetation periods of 1997 to 2000, the mean $NO_2$ concentrations up to a point 22 km northeast of JV Achema varied from 1.94 to 11.48 µg $m^{-3}$, and those of $SO_2$ from 2.49 to 8.45 µg $m^{-3}$ (Table 1). The maximal concentrations of $NO_2$ in the air was 17.16 µg $m^{-3}$ and of $SO_2$ 16.28 µg $m^{-3}$. The annual levels of emissions from JV Achema during the period from 1993 to 1996 failed to exceed the levels found there during 1997 to 2000 (Fig. 1). It can thus be stated that since 1993 the concentrations of pollutants studied in the zone in which tree stands were dam-

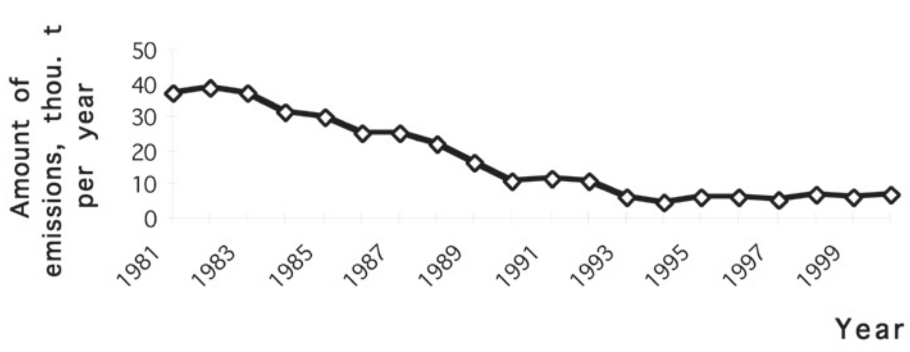

**FIGURE 1.** Changes in emissions from the JV Achema plant during the period from 1981 to 2000.

aged did not exceed the critical annual mean levels found for forest trees in general[17].

## Condition of Scots Pine Stands

In Fig. 2, three stages in the changes in condition of the 50- to 100-year-old Scots pine stands near JV Achema can be distinguished: (1) a worsening until 1992; (2) a stabilization from 1992 to 1995; and (3) an improvement from 1995 to 2000. These stages can be related to air pollution as follows.

- Until 1998, when JV Achema was emitting more than 20,000 tonnes of pollutants annually, tree defoliation in the Scots pine stands nearby increased. When annual emissions fell markedly (to 11,000 to 12,000 tonnes), defoliation continued to increase for another 3 to 4 years. This tendency was shown not by any clear increase in the percentage of mean defoliation (there were no significant differences), but by a significant decrease in the dispersion of the index.
- During a period of 3 to 4 years (from 1992 to 1995) when the air pollution was lower than it had been, defoliation remained stabile.
- After 6 to 7 years of reduced air pollution, refoliation (or a decrease in defoliation) began.

Figure 2 also shows changes since 1989 in the condition of Scots pine stands of analogous age in nonindustrial areas of Lithuania. The mean defoliation of the stands near JV Achema during the period from 1989 to 1999 was higher by some 4 to 12% than in nonindustrial areas. This difference fell markedly (to 2.7%) in the year 2000. It can also be seen that since 1996 an improvement in the condition of the Scots pine stands not only near JV Achema but also in Lithuania as a whole has occurred. It thus appears that the present condition of stands near JV Achema is influenced not only by local air pollution but also by background pollution and by meteorological factors.

## Changes in Ground Vegetation

The deposition of nitrogen compounds can have a double effect on ground vegetation, both that of nutrient enrichment and of acidification. Ground vegetation (grasses, bryophytes, lichens, etc.) is often more sensitive to such effects than are forest trees[18]. In stands in Germany and in the Czech Republic that have been severely damaged by anthropogenic pollution, the observed invasion of nitrophilous plants and grain herbs has been attributed not only to the eutrophication of the environment caused by increased nitrogen deposition[19,20], but also to a reduction in competition due to thinning of the crowns (defoliation), and to weakening of the trees[21]. In the Baltic states anthropogenic phytocenosis is characterized by three interrelated synantrophic processes: ruderalization, graminification, and frutification[22]. Floristic changes most frequently take place due to an abundance of plant species that are not typical of a forest environment. Ellenberg's scale is the indicator most widely employed in Western Europe for describing ecological changes in forest ecosystems that are due to a heightened anthropogenic impact[14,23,24].

According to data obtained in 1999 at a distance of 20 to 22 km from JV Achema, the species composition of the vegetation and the covering of dominant species were then close to those found in such communities in nonindustrial sections of the country[15]. At a reasonably short distance from the plant (10 to 12 km from it) a reduction in the covering of the grass-brush species (*Vaccinium myrtillus*, *V. vitis-idaea)* typical for *Vaccinio-myrtillosum* Scots pine stands, and an increase in the covering of

**TABLE 1**
**Mean Monthly $NO_2$ and $SO_2$ Concentrations (µg $m^{-3}$) in Damaged Forests Near the JV Achema Plant**

| Distance Northeast from the Plant | $NO_2$ | | | | $SO_2$ | | | |
|---|---|---|---|---|---|---|---|---|
| | 1997 | 1998 | 1999 | 2000 | 1997 | 1998 | 1999 | 2000 |
| 0–6 km | 11.48 (6.90–17.16)[a] | 5.50 (0.83–9.74) | 7.83 (1.33–17.52) | 2.71 (1.40–4.90) | 8.45 (3.53–16.28) | 2.62 (0.00–5.40) | 3.48 (1.75–6.36) | 6.39 (0.00–17.20) |
| 8–22 km | 9.38 (7.10–13.04) | 2.68 (0.83–3.92) | 5.46 (3.29–7.38) | 1.94 (1.10–3.20) | 5.59 (2.09–9.14) | 2.49 (0.68–2.74) | 4.54 (1.75–12.74) | 3.86 (0.00–8.05) |
| Critical annual mean levels for forest trees[17] | 30 | | | | 20 | | | |

[a] Minimum and maximum concentrations shown in parentheses.

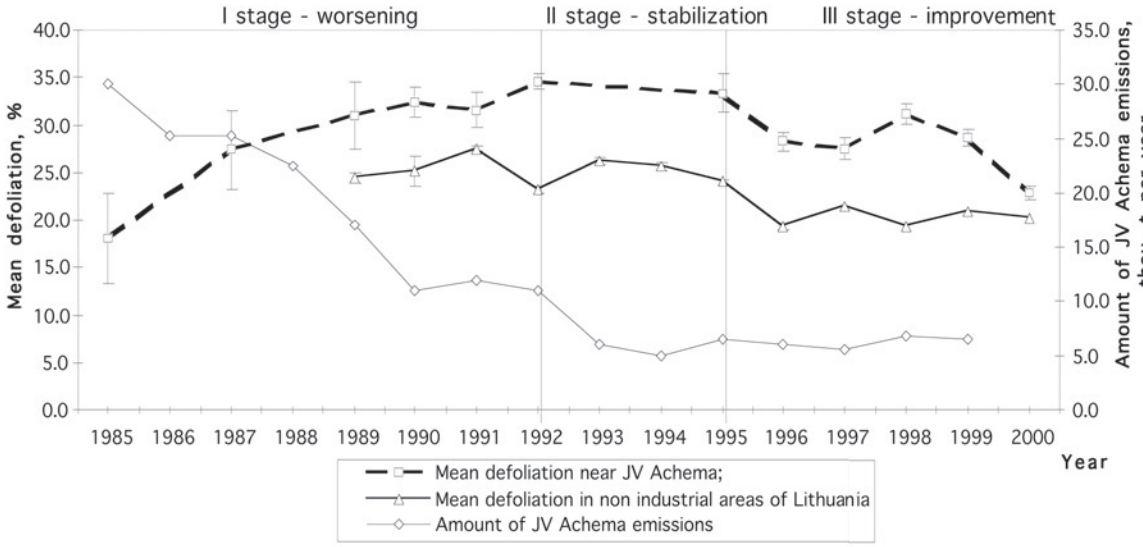

**FIGURE 2.** Mean defoliation in 50- to 100-year-old stands of Scots pine trees 10 to 20 km northeast of the JV Achema plant.

nitrophilous plants (*Rubus idaeus*) could be observed (Table 2). The abundance of mosses characteristic for *Vaccinio-myrtillosum* Scots pine stands also increased as the distance from the plant increased.

The species composition of the vegetation communities and the weighted acid tolerance values ($E_R$) showed that under conditions of reduced air pollution, forest ecosystems closer to the plant (10 to 12 km) remained anthropogenically degraded (Fig. 3). On the other hand, a distinct process of ground vegetation recovery could be observed in terms of an abundance of species typical to *Vaccinio-myrtillosum* Scots pine stands and a reduction in nitrophilous vegetation (Table 2), as well as changes in environ-

**TABLE 2**
**Mean Coverage by the Main Species of *Vaccinio-myrtillosa* of the Forest Type in Scots Pine Stands Near the JV Achema Plant**

| | Distance from Plant | | | |
|---|---|---|---|---|
| | 10–12 km | | 20–22 km | |
| **Species** | **1988** | **1999** | **1988** | **1999** |
| *Chamerion angustifolium* (L.) Holub. | 0.3 ± 0.1[a] | 0.0[a] | 0.0 | 0.0 |
| *Festuca ovina* L. | 4.7 ± 2.5 | 7.6 ± 1.9 | 1.2 ± 0.7 | 5.5 ± 2.8 |
| *Melampyrum prarense* L. | 1.8 ± 0.5 | 3.0 ± 0.7 | 0.7 ± 0.3 | 1.2 ± 0.3 |
| *Rubus idaeus* L. | 24.7 ± 4.0[a] | 2.9 ± 1.5[a] | 4.1 ± 1.7[a] | 0.5 ± 0.2[a] |
| *Vaccinium myrtillus* L. | 5.6 ± 2.2 | 8.8 ± 3.1 | 7.8 ± 1.7[a] | 23.1 ± 5.0[a] |
| *V. vitis-idaea* L. | 1.1 ± 0.4 | 1.5 ± 0.7 | 1.3 ± 0.3 | 3.0 ± 0.9 |
| *Dicranum polysetum* Sw. | 2.7 ± 0.7 | 5.6 ± 1.3 | 9.1 ± 2.4 | 12.2 ± 2.9 |
| *Hylocomium splendens* (Hedw.) Schimp. | 1.6 ± 1.0[a] | 12.2 ± 3.6[a] | 11.6 ± 2.6 | 14.4 ± 3.0 |
| *Pleurozium schreberi* (Brid.) Mitt. | 25.9 ± 3.8[a] | 42.8 ± 5.1[a] | 21.4 ± 3.7[a] | 38.6 ± 6.1[a] |
| *Ptilium crista-castrensis* (Hedw.) De Not. | 2.0 ± 1.1[a] | 13.7 ± 4.1[a] | 1.4 ± 1.0 | 2.3 ± 1.3 |
| Number of sample squares (*n*) | 36 | 40 | 46 | 32 |

[a] Statistically significant ($p = 0.05$) changes between 1988 and 1999.

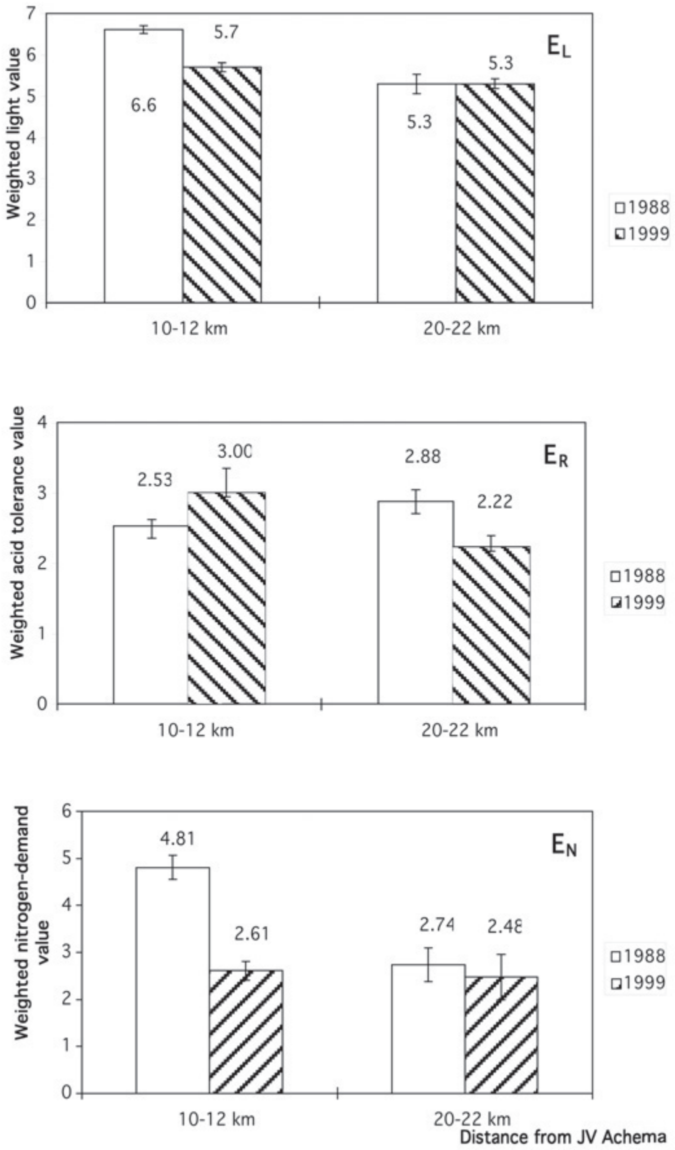

**FIGURE 3.** Changes between 1988 and 1999 in weighted Ellenberg's indicator values ($E_L$ = light, $E_R$ = acid tolerance, $E_N$ = nitrogen-demand) in 60- to 80-year-old stands of *Vaccinio-myrtillosum* (Scots pine) 10 to 12 km and 20 to 22 km northeast of the JV Achema.

mental conditions, as shown by weighted Ellenberg's light ($E_L$) and nitrogen-demand ($E_N$) indicator values (Fig. 3).

## Chemical Changes in Forest Soils

Under conditions of reduced air pollution, changes in the chemical parameters of the mineral horizons for forest soils near JV Achema were found to be influenced by the migration of pollutants from the forest litter, as indicated by studies in clear-cut areas of dead stands in which there was an ongoing mineralization of the forest litter and in Scots pine stands in which changes in the mass of the forest litter were very slight.

In the 20-year period since 1979 the forest litter mass of Luvisols in clear-cut areas of dead Scots pine stands was found to have decreased by a factor of almost 8 (from 67.9 to 8.7 tonnes $ha^{-1}$). Comparison with earlier years showed[5] that in 1996 the accumulation of $NH_4$-N in forest litter had decreased on average by a factor of 56 (as compared with 1989) and that the accumulation of mobile S and $NO_3$-N had decreased by a factor of more than 20. In contrast, the mean changes in the amounts of these chemical compounds in the upper 60 cm of mineral soil were not statistically significant.

Due to the leaching of chemical compounds and to other factors (such as the presence of unexpected pollutants or of unusual meteorological conditions) changes in the acidity of the forest litter, although frequently of considerable weight, are often controversial in terms of how they should be interpreted. As can be seen in Table 3, from 1985 to 1988, when sulphur and nitrogen depositions 0.2 km from the plant (150 kg S $ha^{-1}$ and 120 kg N $ha^{-1}$ annually) exceeded the background level in Lithuania by a factor of at least 9 to 12[5], the mineral horizons of the Luvisols had acidified markedly. During a brief 3-year period, the exchange in acidity in the upper 35 cm of the deep mineral soil layer had increased on average by 0.34 to 0.67 $pH_{KCl}$.

**TABLE 3**
**Mean $pH_{KCl}$ Changes in Luvisols at a 0.2-km Distance from the JV Achema Plant**

| | Depth | | | | | |
|---|---|---|---|---|---|---|
| **Years** | **O (Forest Litter)** | **0–5 cm** | **5–10 cm** | **10–20 cm** | **25–35 cm** | **45–55 cm** |
| 1985 | 4.26 ± 0.27 | 3.50 ± 0.16 | 3.64 ± 0.17 | 4.30 ± 0.14 | 4.19 ± 0.10 | 3.97 ± 0.07 |
| 1987 | 4.68 ± 0.37 | 3.27 ± 0.07 | 3.48 ± 0.09 | 3.79 ± 0.19 | 4.10 ± 0.11 | 4.19 ± 0.06 |
| 1988 | 4.14 ± 0.48 | 3.01 ± 0.25 | 3.30 ± 0.11 | 3.63 ± 0.12 | 3.85 ± 0.21 | 3.89 ± 0.10 |
| 1989 | 4.83 ± 0.31 | 3.38 ± 0.05 | 3.48 ± 0.08 | 3.85 ± 0.16 | 4.03 ± 0.09 | 4.04 ± 0.19 |
| 1992 | 5.04 ± 0.32 | 3.36 ± 0.03 | 3.38 ± 0.03 | 3.44 ± 0.33 | 4.08 ± 0.05 | 3.96 ± 0.09 |
| 1998 | 5.63 ± 0.27 | 3.47 ± 0.09 | 3.40 ± 0.04 | 3.55 ± 0.09 | 4.03 ± 0.19 | 4.05 ± 0.06 |
| 1999 | 4.84 ± 0.14 | 3.54 ± 0.11 | 3.60 ± 0.06 | 3.86 ± 0.06 | 4.13 ± 0.07 | 4.15 ± 0.09 |
| 2000 | 5.22 ± 0.40 | 3.82 ± 0.12 | 3.96 ± 0.10 | 4.19 ± 0.10 | 4.45 ± 0.08 | 4.46 ± 0.07 |
| $\Delta pH_{1985\text{-}1988}$ | –0.12 | **–0.49**[a] | **–0.34**[a] | **–0.67**[a] | **–0.34**[a] | –0.08 |
| $\Delta pH_{1988\text{-}1999}$ | **+0.70**[a] | **+0.53**[a] | **+0.30**[a] | **+0.23**[a] | +0.28 | **+0.26**[a] |
| $\Delta pH_{1999\text{-}2000}$ | +0.38 | **+0.28**[a] | **+0.36**[a] | **+0.33**[a] | **+0.32**[a] | **+0.31**[a] |

*Note:* Means ± s.e. ($n$ = 10) are shown.

[a] Statistically significant ($p$ = 0.05) changes.

When air pollution decreased, the reaction of forest litter became more alkaline, although the acidity of the mineral horizons remained unchanged, only beginning to decrease after 1998 (Fig. 4).

In terms of the data presented, it appears that changes in forest soil acidity proceed rather slowly during a reduction in air pollution. One can thus suggest that forest soil should be monitored at least every 10 years[13]. During a similar 10- to 12-year period (1988 to 1999) in almost all cases at locations near to JV Achema, Luvisols were found to become 0.23 to 0.70 $pH_{KCl}$ more alkaline (Table 3). This process is now even more intensive. During a period of only a year (1999 to 2000), the acidity of the upper 55-cm layer of Luvisols was found to decrease by 0.28 to 0.36 $pH_{KCl}$.

In comparing Figs. 3 and 4, one can note that when air pollution was reduced the recovery reaction (the decrease in acidity) of the studied Luvisols was more gradual and started 3 to 4 years later than the refoliation of the Scots pine stands.

The changes in the chemical parameters of the Arenosols in the Scots pine stands that survived when air pollution was reduced were significant as well (Fig. 5). Although their reaction did not change, from between 1987 and 1989 to 1999, over 10 to 12 years, mobile S leached (amounts were less than the analysis precision limit) from forest litter of polluted Arenosols; in addition, quantities of $NO_3$-N decreased on average 5.5 times, $P_2O_5$ and $Al^{3+}$ about 2 times, and $NH_4$-N 1.7 times.

In the forest litter of the unpolluted Arenosols (controls), there was a marked decrease in the amounts of mobile S by a

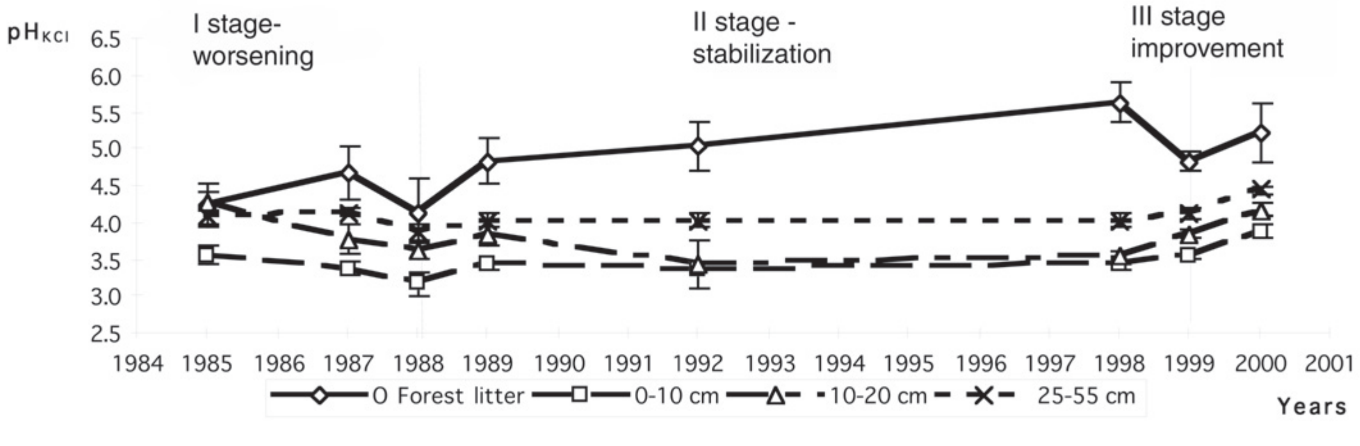

**FIGURE 4.** Mean $pH_{KCl}$ changes in Luvisols at a distance of 0.2 km from the JV Achema plant during the period from 1985 to 2000.

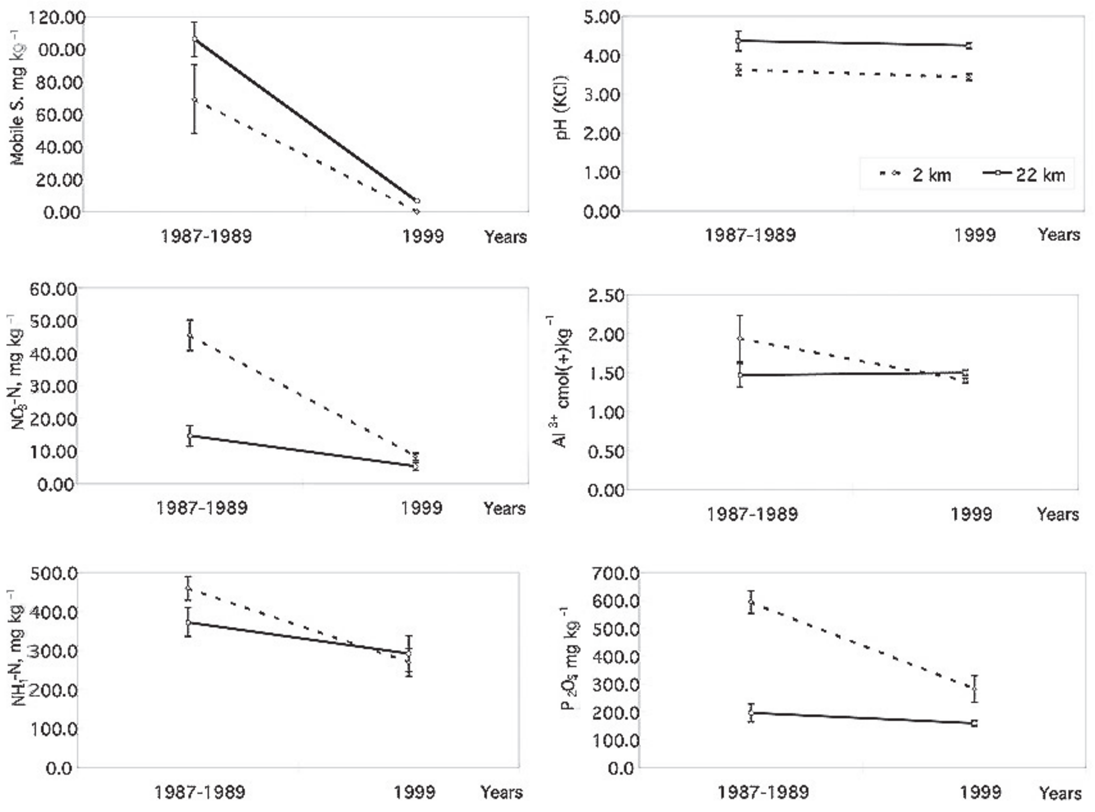

**FIGURE 5.** Changes between 1987 to 1989 and 1999 in chemical parameters of forest litters of polluted (2 km) and unpolluted (22 km) Arenosols in Scots pine stands near JV Achema plant.

factor of 16 on average, and of $NO_3$-N by a factor of 2.8. This can be attributed to the reduction in background pollution both in Europe in general and in Lithuania[25].

Summarizing the data in Fig. 5, one can state that in 1999, when the air pollution had been reduced, the contamination of the forest litter of the Arenosols had diminished markedly as well. The amounts of mobile N forms, of $P_2O_5$, and of $Al^{3+}$ became statistically similar to those of the controls, the amounts of mobile S in them being even less. However, the forest litter of polluted Arenosols was on the average 0.81 $pH_{KCl}$ more acidic.

As Fig. 6 indicates, the amounts of $NO_3$-N and mobile S underwent the greatest changes in the mineral horizons of the polluted Arenosols over a 10- to 12-year period. In the entire profile studied, the amounts of $NO_3$-N decreased on average at a depth of 0 to 20 cm by as much as 20 to 34 times, and at a depth of 25 to 55 cm by 4 to 5 times. This should be interpreted as a decrease in the nitrification (formation of nitric acid) that was stimulated earlier by the high ammonia deposition that contributed to an increase in pH of the organic layer (Fig. 4). When mobile S in the upper 10-cm deep layer was leached out, its amount in deeper layers remained statistically unchanged. Changes in the amounts of $NH_4$-N and $P_2O_5$ were in almost all cases not statistically significant (at a $p = 0.05$ level). The chemical parameters ($pH_{KCl}$ and the amount of $Al^{3+}$) that reflected acidification of the mineral horizons of the polluted Arenosols did not change significantly over a period of 10 to 12 years (Fig. 7). This is similar to the results seen from European roof trials to exclude acid deposition[26].

In the control Arenosols, in contrast to the polluted ones, of all the mineral soil chemical parameters that were studied it was only the amounts of $NO_3$-N, that changed significantly over a 10- to 12-year period (Figs. 6 and 7). These parameters decreased in most cases by a factor of 2 to 4.

## CONCLUSIONS

During the reduction in air pollution by $NO_x$, $NH_3$, $SO_2$, and mineral dust over the last 10 years, an obvious recovery of the damaged forest ecosystems near the nitrogen fertilizer plant JV Achema was observed. After the air pollution had decreased, defoliation of the 50- to 100-year-old Scots pine stands was found to have stabilized or to still be in progress. Refoliation of the damaged Scots pine stands began some 6 to 7 years later. But even when air pollution had decreased, ground vegetation in the Scots pine stands remained anthropogenically degraded. Although a distinct recovery process could be observed, an enrichment of typical *Vaccinio-myrtillosum* forest-type species and a reduction in the number of nitrophilous plants was evident. Changes in the

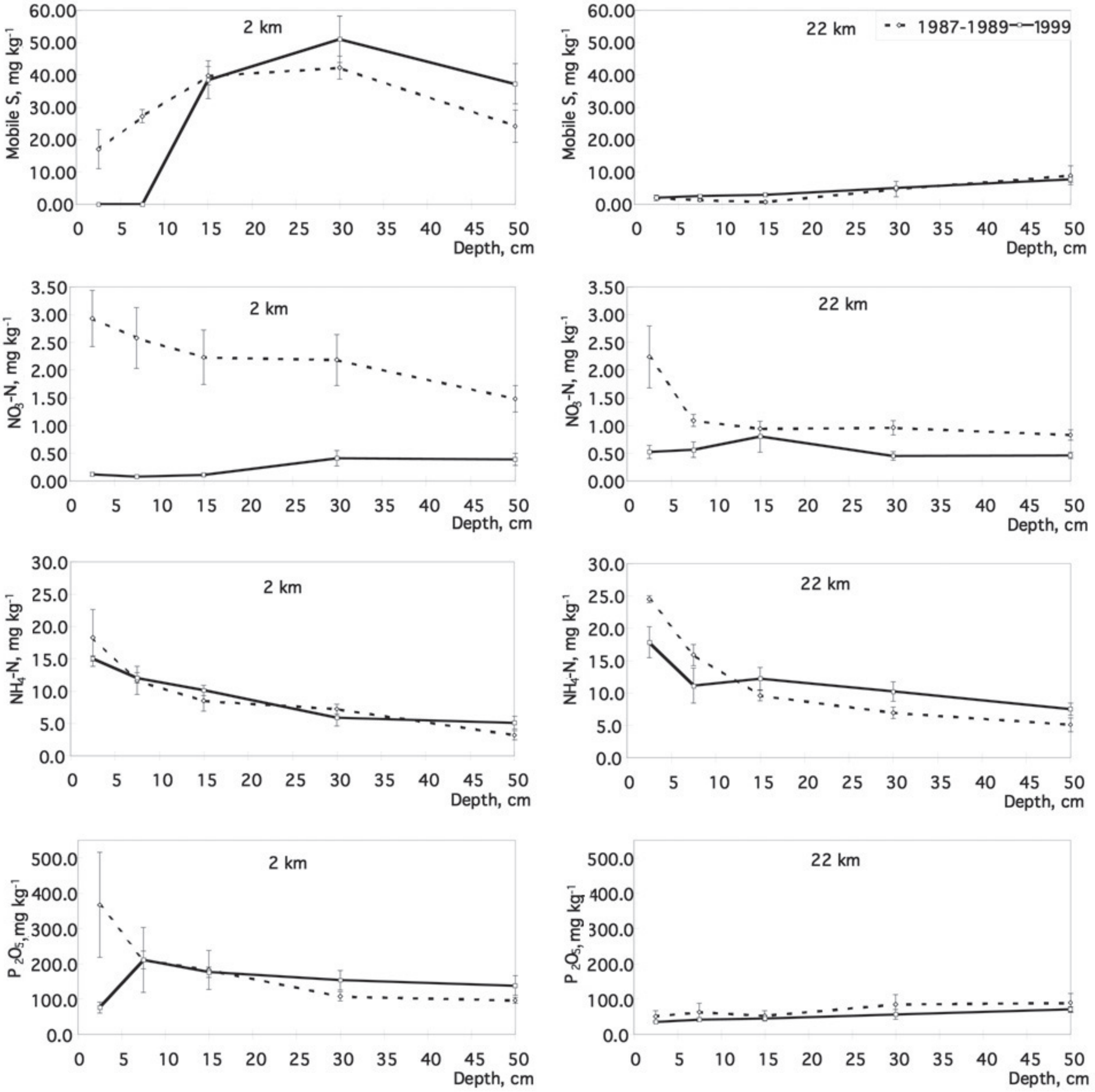

**FIGURE 6.** Changes between 1987 to 1989 and 1999 in the accumulation of pollutants in the upper mineral horizons of polluted (2 km) and unpolluted (22 km) Arenosols near JV Achema plant.

chemical parameters of the mineral horizons of polluted forest soils were influenced by pollutant migration from the forest litter. In clear-cut areas of dead stands in which there was ongoing mineralization of the forest litter and nitrification of deposited ammonia/ammonium a decrease in the acidity of Luvisols, as compared with the refoliation of the Scots pine stands, started 3 to 4 years later. In the Scots pine stands, contamination of the forest litter by mobile S, N, and P was significantly reduced, with no change in mass. And although the amounts of $NO_3$-N and of mobile S diminished in the mineral horizons of the polluted Arenosols, their acidity had not decreased.

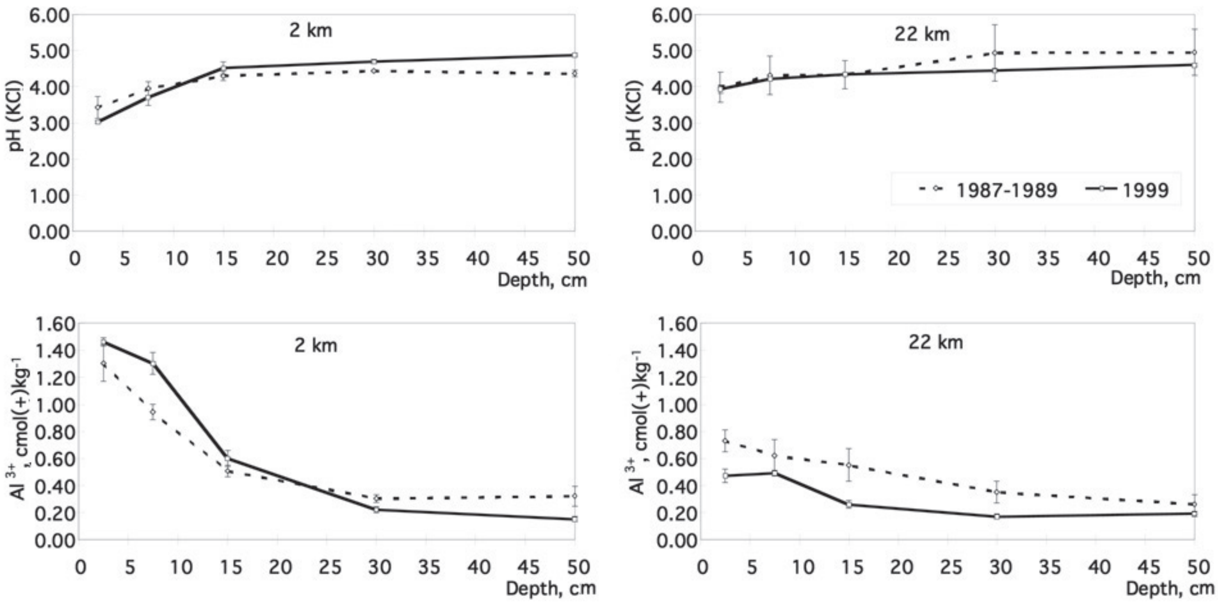

**FIGURE 7.** Acidity changes between 1987 to 1989 and 1999 in the upper mineral horizons of polluted (2 km) and unpolluted (22 km) Arenosols near JV Achema plant.

## ACKNOWLEDGEMENTS

This study was funded by the Lithuanian State Science and Studies Fund, the Jonava district municipality, and by JV Achema. We are grateful to Professor Stasys Karazija for the field data on the ground vegetation in 1988 that he provided to us.

| | | |
|---|---|---|
| Received: | July | 11, 2001 |
| Revised: | November | 14, 2001 |
| Accepted: | November | 14, 2001 |
| Published: | December | 8, 2001 |

## BIOSKETCHES

**Kestutis Armolaitis** is Head of the Department of Forest Soils, Typology and Hydrology of the Lithuanian Forest Research Institute (Kaunas-Girionys, Lithuania), and part-time associate professor at Vytautas Magnus University in Kaunas. Dr. Armolaitis graduated from the Forest Faculty of Lithuanian Agricultural University in Kaunas in 1977 and was awarded the degree of doctor of silvicultural sciences in the Technological University of Byelorussia in Minsk in 1984. In 1992 he was on probation at the Department of Silviculture of Swedish University of Agricultural Sciences (Umeå). His research interests include regional and local symptoms and consequences of forest ecosystems decline due to air pollution by $NO_x$, $NH_3$, $SO_2$ and heavy metals; assessment and biotesting of changes in chemical parameters of contaminated forest soils; recovery and management of damaged forests; and scientific background of nation-wide and local monitoring of forest ecosystems.

**Vidas Stakenas** is Senior Scientist of the Department of Forest Ecology and Silviculture at the Lithuanian Forest Research Institute. Dr. Stakenas graduated from the Faculty of Natural Sciences of Vilnius State University in 1980. In 1993 he was awarded the degree of doctor of silvicultural sciences in the Lithuanian Forest Research Institute. His research interests include the scientific background of monitoring of forests ecosystems; methods of trees and forest ecosystems condition assessment; influence of climatic factors on tree crown condition; photosynthetical active radiation; leaf area index (direct and indirect methods); changes of ground vegetation species composition due to natural and anthropogenical factors; and phytoindication.
